# Efficacy and Safety of a Traditional Chinese Herbal Formula Xuefu Zhuyu Decoction for Hypertension: A Systematic Review and Meta-Analysis

**DOI:** 10.1097/MD.0000000000001850

**Published:** 2015-10-23

**Authors:** Pengqian Wang, Xingjiang Xiong, Shengjie Li

**Affiliations:** From the Institute of Basic Research in Clinical Medicine, China Academy of Chinese Medical Sciences, Beijing, China (PW); Department of Cardiology, Guang’anmen Hospital, China Academy of Chinese Medical Sciences, Beijing, China (XX); and Department of Biological Science and Technology, School of Life Sciences, Tsinghua University, Beijing, China (SL).

## Abstract

The cardioprotective role of xuefu zhuyu decoction (XZD), a well-known classical herbal formula, has been documented for hypertension treatment recently. This study aims to summarize the efficacy and safety of XZD in treating hypertension.

Seven databases were searched to identify randomized controlled trials evaluating the efficacy of XZD in hypertensive patients. Fifteen studies involving 1364 hypertensive patients were included. All studies compared XZD and antihypertensive drugs with antihypertensive drugs used alone.

In all, 15 studies reported significant effects of XZD for lowering blood pressure compared with the control group (*P* < 0.05), and 7 studies reported significant effects of XZD for improving symptoms compared with the control group (*P* < 0.00001). Meanwhile, studies reported XZD was more efficacious than antihypertensive drugs in improving total cholesterol, triglycerides, low-density lipoprotein cholesterol, homocysteine, hemorheology, carotid intima-media thickness, and left ventricular mass index (*P* < 0.05). No severe adverse event was reported.

This meta-analysis provides evidence that XZD is beneficial for hypertension. Although concerns regarding selective bias and methodologic flaws were raised, our findings suggests XZD as a new candidate cardioprotective drug for hypertension, which should be given priority for future preclinical and clinical studies.

## INTRODUCTION

Hypertension is defined as a systolic blood pressure (SBP) of ≥140 mm Hg or a diastolic blood pressure (DBP) of ≥90 mm Hg and/or the current use of antihypertensive medication.^1^ Epidemiologic surveys have identified a strong association between hypertension and cardio- and cerebrovascular diseases.^2,3^ The estimated number of the affected world's adult population was 26.4% (972 million) in 2000, and the rates are expected to increase to 29.2% (1.56 billion) by 2025.^4^ It has become a major contributor to death and disability from heart and vascular diseases. Antihypertensive therapy, especially when combined with effective lipid-lowering therapy, reduces the cardiovascular morbidity and mortality rates^5–8^; however, the current status of treatment is unsatisfactory.^9,10^ Hence, additional therapeutic approaches with comparatively few adverse effects are gaining increasing popularity worldwide.^11–14^

Since the publication of Scientific Statement on Alternative Approaches to Lowering Blood Pressure by American Heart Association^15^ and Clinical Expert Consensus Documents on Integrating Complementary Medicine Into Cardiovascular Medicine by American College of Cardiology,^16^ there has been growing clinical interests in the benefits, harm, and potential herb–drug interactions of complementary and alternative medicine (CAM) for hypertension, including qigong,^17^ tai chi,^18^ baduanjin exercise,^19^ yoga,^20^ massage,^21^ acupuncture,^22^ moxibustion,^23^ cupping,^24^ dietary supplements,^25^ and herbal medicine products.^26^ As one of the most important components of CAM, traditional Chinese medicine (TCM) has been used for thousands of years and is still being widely practiced.^27,28^ The study of Chinese herbal formulae for promoting blood circulation and removing blood stasis (PBCRBS) for cardiovascular diseases is the active area of research focus within TCM and integrative medicine in East Asia.^29–31^ Recently, Chinese herbal medicine for PBCRBS as a CAM approach has been well recognized in treating hypertension.^32,33^ The current evidence of some traditional Chinese patent medicine for PBCRBS, which have been approved by China Food and Drug Administration for hypertension, was also summarized for clinical recommendations.^34–36^ Thus, PBCRBS-based Chinese herb and formulae have been exploited as an important therapy for hypertension.

Xuefu Zhuyu Decoction (XZD), a well-known PBCRBS-based traditional Chinese classical herbal formula, is recorded in the medical classic *Yi Lin Gai Cuo* by the Chinese physician Wang Qingren (1768–1831) approximately 200 years ago.^37^ The multiple cardiovascular protective actions of XZD with no adverse effects have been documented recently.^38–40^ It is efficient in lowering blood pressure (BP) and alleviating BP-related symptoms caused by qi stagnation and blood stasis syndrome according to TCM theory.^41^ XZD is composed of 11 Chinese herbs: Peach Kernel (Taoren, Persicae Semen), Safflower Flower (Honghua, Flos Carthami Tinctorii), Chinese Angelica Root (Danggui, Radix Angelicae Sinensis), Rehmannia (Di Huang, Radix Rehmanniae Glutinosae), Szechuan Lovage Root (Chuanxiong, Rhizoma Ligustici Chuanxiong), Red Peony Root (Chi Shao, Radix Rubrus Paeoniae Lactiflorae), Achyranthes Root (Niu Xi, Achyranthis Bidentatae Radix), Root of the Balloon Flower (Jiegeng, Platycodi Radix), Thorowax Root (Chaihu, Radix Bupleuri), Orange Fruit (Zhike, Fructus Aurantii), and Liquorice Root (Gan Cao, Radix Glycyrrhizae), with 5-hydroxymethyl-2-furaldehyde, hydroxysafflor yellow A, amygdalin, albiflorin, paeoniflorin, liquiritin, ferulic acid, naringin, hesperidin, neohesperidin, isoliquiritigenin, and glycyrrhizic acid as the major active compounds.^42^ The mechanism of XZD for hypertension lies in inhibition of renin–angiotensin–aldosterone system,^43^ improvement of endothelial function and prethrombotic state,^44^ inhibition of vascular remodeling,^45,46^ and prevention of myocardial fibrosis.^47–49^ Numerous clinical trials have been published reporting the beneficial effects of XZD for hypertension in China; however, no systematic review specifically addressing XZD has been conducted. Thus, a systematic review and meta-analysis of the current available randomized controlled trials (RCTs) was considered appropriate and timely. Given this background, this study aims to comprehensively examine the efficacy and safety of XZD for hypertension.

## METHODS

This systematic review is conducted in accordance with the Preferred Reporting Items for Systematic Reviews and Meta-Analyses: The PRISMA Statement.^50^

## ELIGIBILITY CRITERIA

### Types of Studies

We only included RCTs in this systematic review, regardless of blinding, publication status, or language. Animal studies were not considered.

### Types of Participants

Only hypertensive patients were included. No restriction on sex, age, or ethnicity was predefined. Hypertension should be diagnosed clinically according to the criteria documented in the seventh report of the Joint National Committee or other guidelines and definitions.^1^

### Types of Interventions

RCTs that examined the effect of XZD either used alone or in combination with western medicine comparing with placebo, no treatment or western medicine were identified. Participants in the treatment group should be treated by XZD-based formula or XZD combined with western medicine. Participants in the control group should be treated by placebo, no treatment or western medicine. The western medicine used in the treatment group should be the same as the controls in the category, dosage and method of administration. Studies were excluded if other CAM therapies beyond Chinese herbal medicine, including yoga, Tai Chi, qigong, acupuncture, moxibustion, cupping and massage, were used in either the treatment group or control group; if other Chinese herbal medicine therapies were used in the control group; if the efficacy of XZD on BP outcome measure was not reported; and if duplicate publication reporting the same conclusions were identified. The definition of XZD-based formula is XZD used alone or the modified XZD based on TCM theory. We have not set any restriction on blinding and treatment duration.

### Types of Outcome Measures

As antihypertensive therapy is the cornerstone of hypertension treatment, the primary outcome measures were defined as SBP, DBP, and categorical BP at the end of the treatment course. China Food and Drug Administration has adopted 3 classifications to evaluate the therapeutic effects of TCM on categorical BP, which was documented in the Guidelines of Clinical Research of New Drugs of Traditional Chinese Medicine (GCRNDTCM). They were as follows: (1) significant improvement—DBP decreased by 10 mm Hg and reached the normal range; (2) improvement—DBP decreased by <10 mm Hg but reached the normal range; and (3) no improvement—BP was not decreased.^51^ The secondary outcome measurements were defined as symptoms, blood lipids, homocysteine (HCY), hemorheology, carotid intima-media thickness (IMT), left ventricular mass index (LVMI), and adverse events.

### Search Strategy

Relevant publications were electronically searched in 7 databases: Cochrane Library (1996–May 2015), PubMed (1959–May 2015), Embase (1966–May 2015), Chinese Biomedical Literature Database (1978–May 2015), Wanfang database (1985–May 2015), VIP Information Database (1989–May 2015), and China National Knowledge Infrastructure (1979–May 2015). We also manually searched the references of identified studies and ongoing registered clinical trials to retrieve unpublished articles. No restriction on publication language and status was preset. The following search terms were used: (“high blood pressure” OR “hypertension” OR “blood pressure” OR “gao xue ya” OR “xue ya”) AND (“xuefu zhuyu decoction” OR “xuefu zhuyu tang”) AND (“clinical trial” OR “randomized controlled trial” OR “randomised controlled trial” OR “lin chuang yan jiu” OR “lin chuang shi yan”).

### Study Selection

The titles and abstracts of all the selected articles were independently screened by 2 reviewers according to the eligibility criteria listed above. Duplicate publications were removed accordingly. Then, full texts of potentially relevant articles were retrieved for further assessment. Disagreements were resolved by consultation with a third reviewer.

### Data Extraction

Basic information of the eligible studies were extracted by 2 reviewers independently using a standardized data extraction form. The extracted details included the following: (1) basic information of the studies—title, authors’ name, and publication time; (2) basic characteristics of the enrolled patients—age, sexuality, sample size, diagnosis criteria of hypertension and TCM syndrome, baseline difference, and BP before the treatment; (3) basic characteristics of the studies—methodologic quality, interventions in the treatment and control groups, compositions, dosage and administration methods of XZD-based formula, intention-to-treat analysis, and treatment duration; and (4) primary and secondary outcome measures—SBP, DBP, categorical BP, symptoms, blood lipids, HCY, hemorheology, IMT, LVMI, and adverse events. The correspondence authors of the included studies were contacted by e-mail, fax, and telephone number to obtain the missing data.

### Quality Assessment

The methodologic quality of the eligible trials was assessed using the Cochrane Collaboration's tool.^52^ The criteria from the Cochrane Handbook for Systematic Reviews of Interventions is composed of the following 8 items: (1) adequate sequence generation; (2) concealment of allocation; (3) blinding of the patient; (4) blinding of the investigator; (5) blinding of the assessor; (6) incomplete outcome data addressed (intention-to-treat analysis); (7) free of selective reporting; and (8) other potential threat to validity. Two reviewers independently conducted the quality assessment. The third party was consulted if disagreements were identified.

### Data Synthesis

Comparison between XZD and antihypertensive drugs (XPAD) and antihypertensive drugs alone was performed in this review. Outcome measures after treatment were presented as weighted mean difference (WMD) with 95% confidence interval (CI) for continuous outcomes, and risk ratio (RR) with 95% CI for dichotomous outcomes. Heterogeneity of effect sizes was tested using the *I*^*2*^ statistics. A random-effects model was adopted to assess the effects of XZD-based formula for hypertension across trials if substantial heterogeneity was observed (*I*^*2*^ > 50% or *P* < 0.1); otherwise, a fixed-effects model was used. A funnel plot was used to examine the publication bias. *P* < 0.05 was considered to be statistically significant. All of data in this meta-analysis were synthesized using the Review Manager software (RevMan, Version 5.3, Copenhagen: The Nordic Cochrane Centre, The Cochrane Collaboration, 2014).

## RESULTS

### Study Selection

**FIGURE 1:**
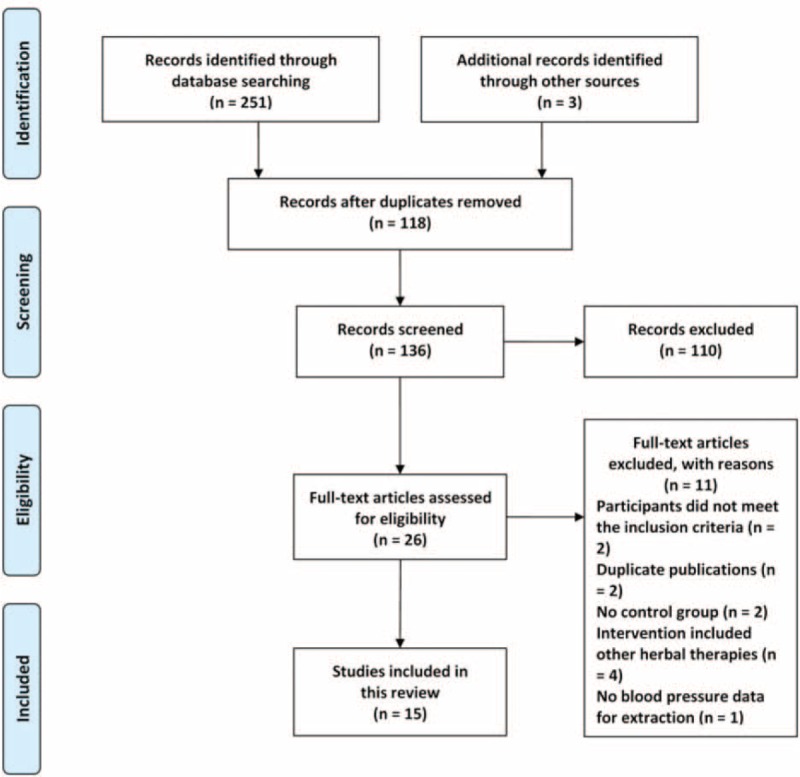
Flow diagram of study selection and identification.

Among the 254 studies identified in the literature search, 118 duplicate publications were excluded. After reading the titles and abstracts, 110 articles were excluded because they were case studies, case series, animal experiments, or nonhypertensive patients. Then, 26 full-text articles were assessed for eligibility and we excluded 11 trials because of the following reasons: 2 articles did not meet the inclusion criteria; 2 articles were duplicate publications; 2 articles had no control groups; intervention in 4 articles included other herbal therapies; and 1 article had no BP data for extraction. Ultimately, 15 eligible studies involving a total of 1364 patients with hypertension were identified in the review.^53–67^ The flow diagram of study selection and identification was summarized in Figure 1.

### Study Characteristics

The descriptive information of the included trials and subjects in this review was summarized in Tables 1 and 2. All of 15 trials were conducted in a single center of China and published in Chinese between 2001 and 2015. The sample size ranged from 60 to 128 with a mean size of 91. All patients enrolled were diagnosed as hypertension, which was based on criteria of World Health Organization-International Society of Hypertension Guidelines for the Management of Hypertension-1999,^53–55,59,63,64,66,67^ Chinese Guidelines for the Management of Hypertension-2010 (CGMH-2010),^56,65^ GCRNDTCM,^57^ and Chinese Guidelines for the Management of Hypertension-2005 (CGMH-2005).^58,60–62^ The diagnostic criteria of TCM syndrome was reported in 10 trials, including GCRNDTCM,^53,54,56–58,61,65^ Traditional Chinese Medicine-Syndrome Differentiation Criteria (TCM-SDC),^55,66^ and Guidelines for Diagnosis and Treatment of Common Internal Diseases in Chinese Medicine-2008 (GDTCIDCM-2008).^62^ The age of the enrolled hypertensive patients ranged from 31 to 83 years old. No significant difference on baseline was identified in all the studies. All trials compared XZD with no treatment control, that was, XPAD versus antihypertensive drugs. Treatment duration ranged from 10 days to 24 weeks. One trial reported the dropouts^61^ and no trial reported source of funding. Interventions of XZD and antihypertensive drugs were all given orally. The dosage of XZD was 1 dose/d in all trials. The components of XZD-based formula in each study were depicted in Table 3  . BP outcomes were reported in all the enrolled studies, with continuous BP in 9 trials^53–61^ and categorical BP in 6 trials.^62–67^ The symptoms outcomes were reported in 7 trials.^55–57,59,61,65,67^ The outcomes of blood lipids were reported in 4 trials.^53,56,57,66^ The serum HCY level was reported in 1 trial.^60^ The outcomes of hemorheology were reported in 2 trials.^57,61^ The outcome of IMT was reported in 1 trial.^53^ The LVMI outcome was reported in only 1 trial.^58^ Adverse events were reported in 3 trials.^57,59,61^

**TABLE 1:**
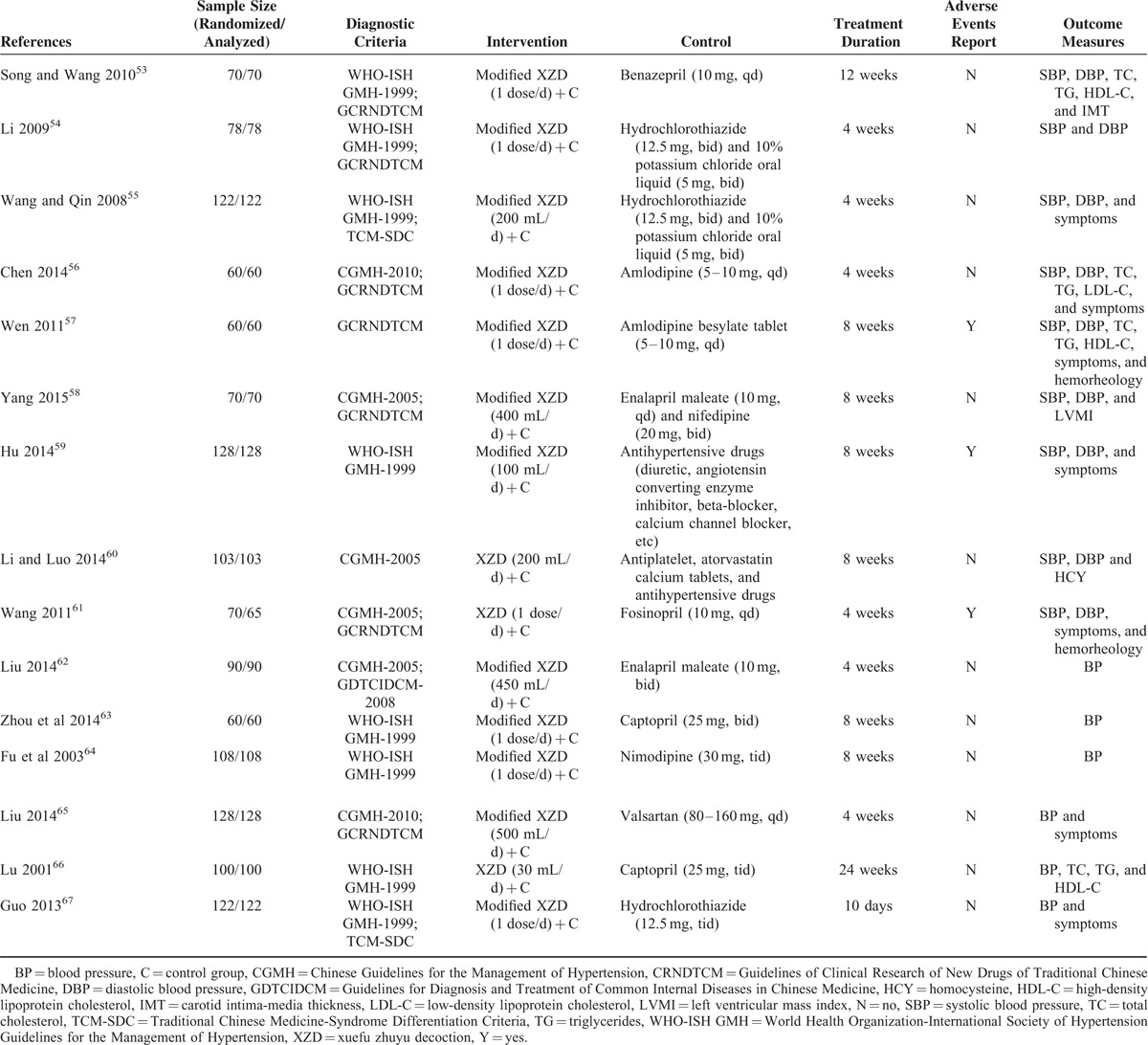
Basic Characteristics of the Included Trials

**TABLE 2:**
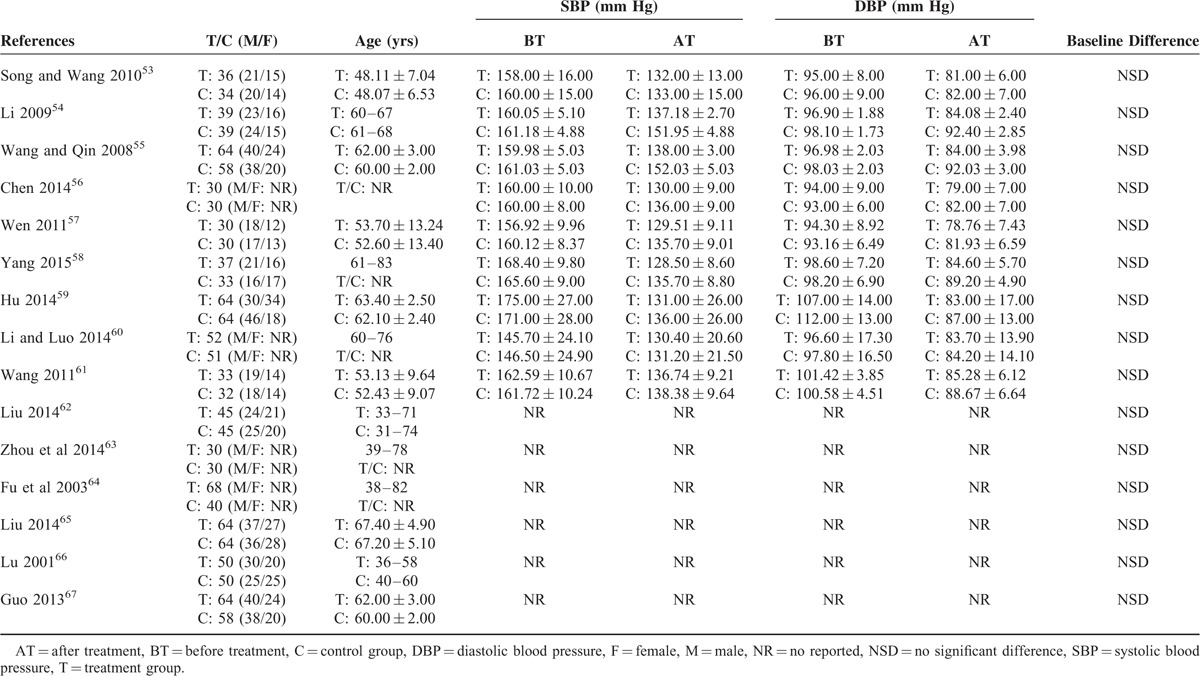
Basic Characteristics of the Included Subjects

**TABLE 3:**
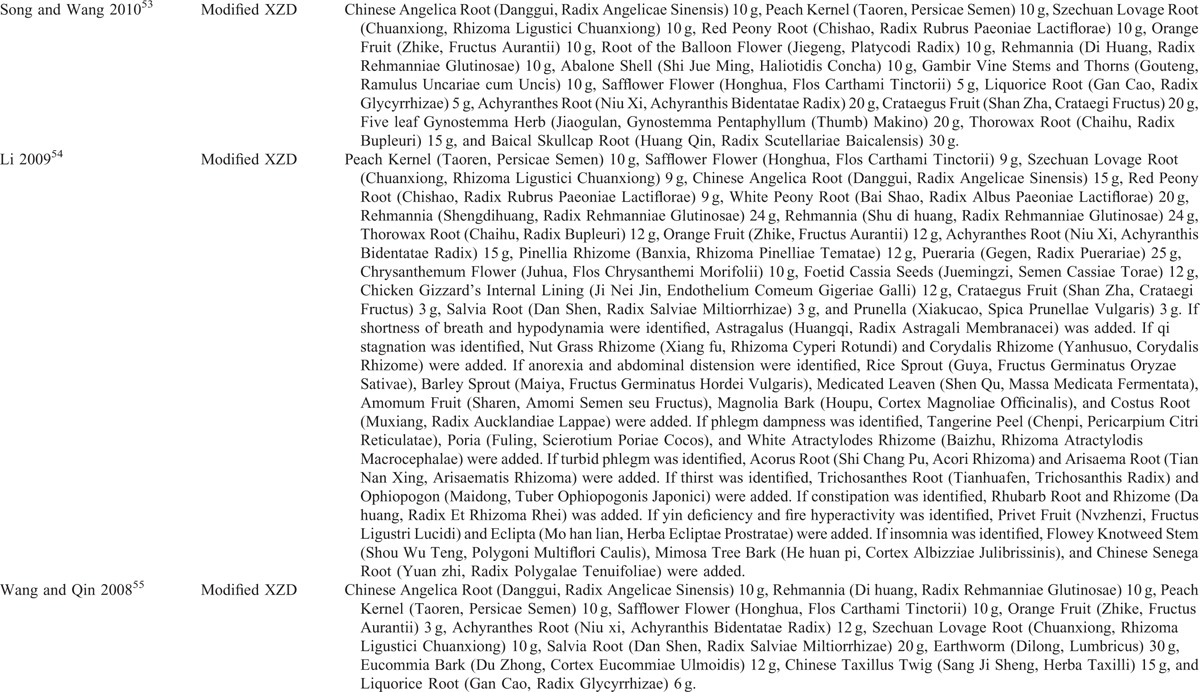
Components of Chinese Herbal Medicine Used in the Included Trials

**TABLE 3 (Continued):**
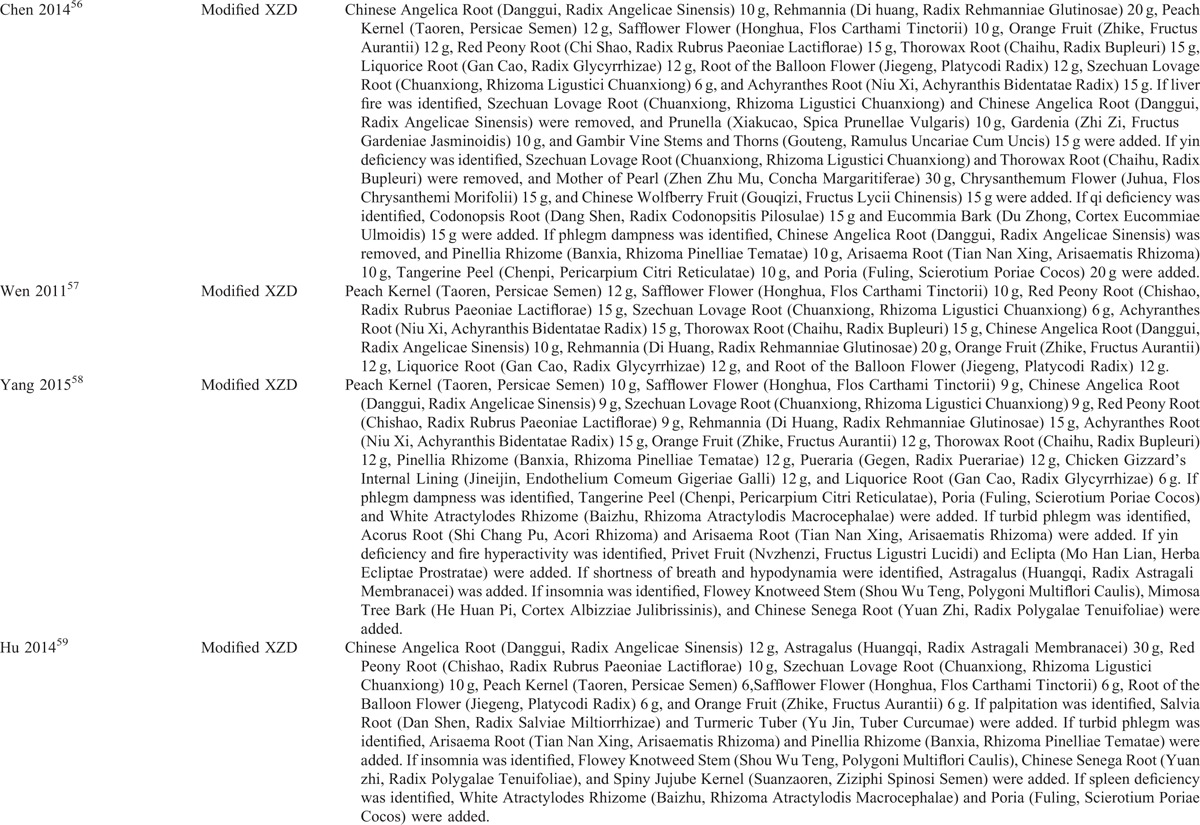
Components of Chinese Herbal Medicine Used in the Included Trials

**TABLE 3 (Continued):**
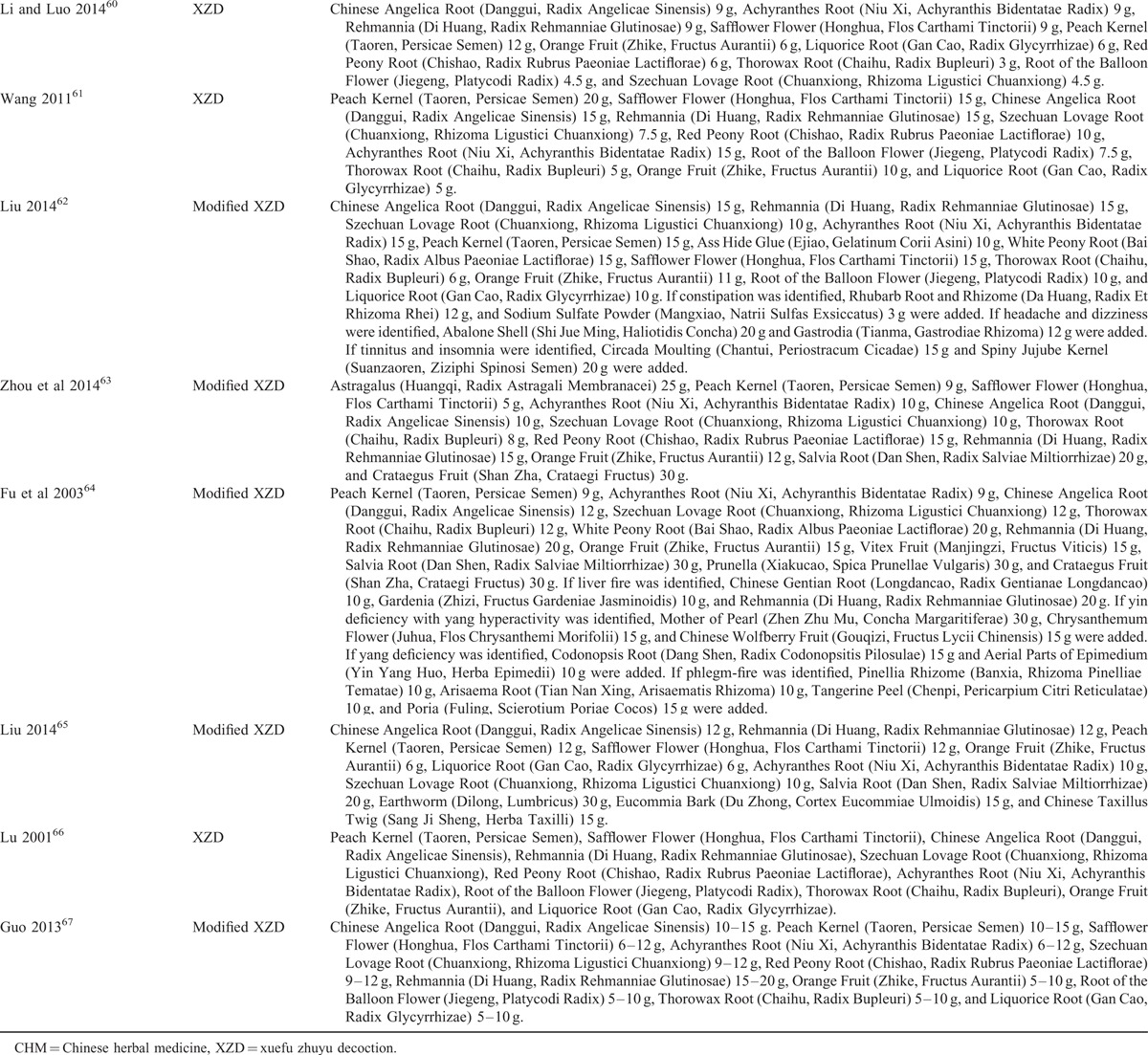
Components of Chinese Herbal Medicine Used in the Included Trials

### Methodologic Quality

The assessment of methodologic quality of each included trial was summarized in Table 4. Among them, 5 trials declared how to generate the random sequence^58,59,61,62,65^; however, the other 10 trials only mentioned randomization in the text without detailed information. Details regarding concealment of allocation and blinding of patient, investigator and assessor were unclear in all the studies. One trial provided the number and reasons of dropouts^61^ and the other 14 trials reported that all the enrolled subjects had completed the trial; however, both selective reporting and other potential threat to validity can not be assessed due to insufficient information provided in the original trials. Additionally, no study reported the methods of sample size calculation and follow-up.

**TABLE 4:**
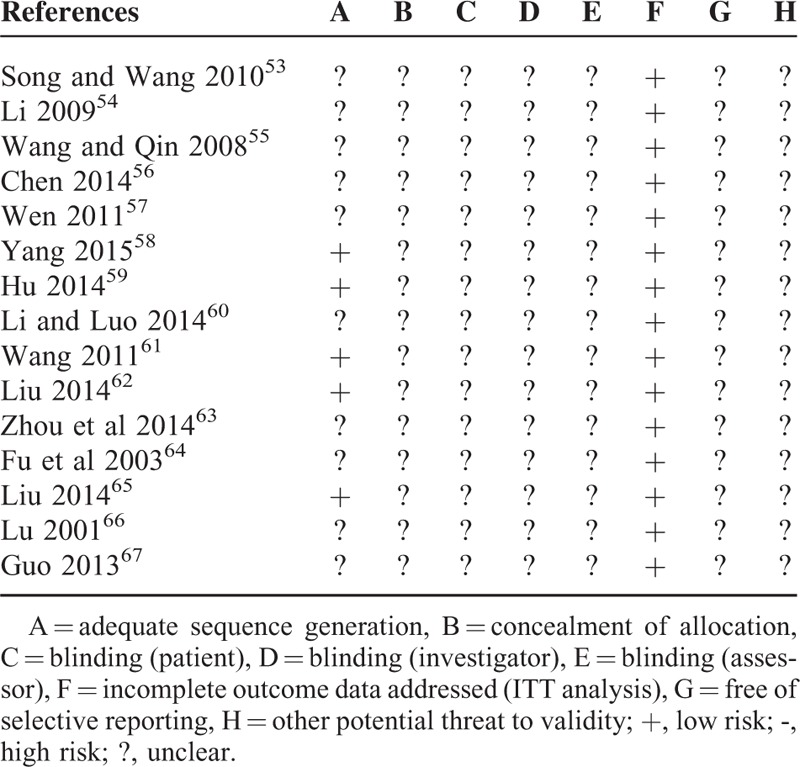
Methodologic Quality of the Included Trials Based on the Cochrane Handbook

## OUTCOME MEASURES

### BP

**FIGURE 2:**
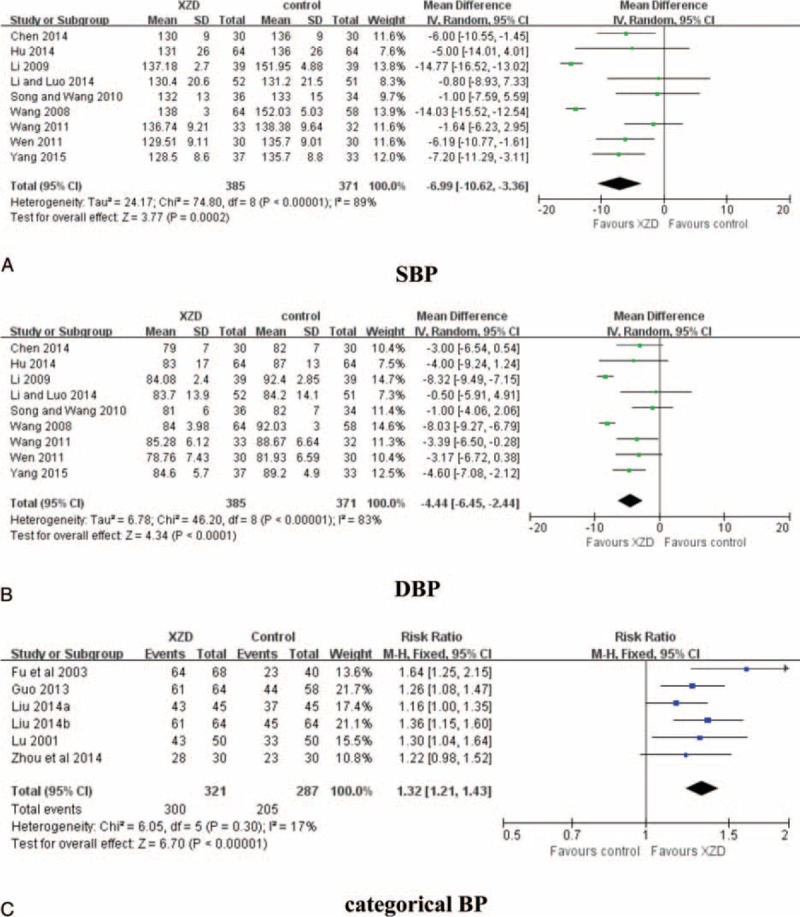
Forest plot of the comparison of XZD versus antihypertensive drugs for the outcome of BP. A, SBP; B, DBP; and C, categorical BP. BP = blood pressure, DBP = diastolic blood pressure, SBP = systolic blood pressure, XZD = xuefu zhuyu decoction.

The effectiveness of XZD on BP was evaluated in all of the 15 trials. Continuous BP was used in 9 trials in this meta-analysis.^53–61^ There were 385 patients in the XZD groups and 371 patients in the antihypertensive drugs groups, respectively. A random-effects model was used for statistical analysis according to the test of heterogeneity (SBP: chi-square = 74.80, *P* < 0.00001, *I*^2^ *=* 89%; DBP: chi-square = 46.20, *P* < 0.00001, *I*^2^ *=* 83%). The combined effects of these 9 independent trials showed a significant lowering effects of XZD on SBP (WMD = −6.99 mm Hg; 95% CI: −10.62 to −3.36, *P* = 0.0002) and DBP (WMD = −4.44 mm Hg; 95% CI: −6.45 to −2.44, *P* < 0.0001) in patients with hypertension when compared with antihypertensive drugs alone (Fig. 2A and B). Categorical BP was used in the other 6 trials to evaluate the efficacy of XZD.^62–67^ There were 321 patients in the XZD groups and 287 patients in the antihypertensive drugs groups, respectively. The categorical BP data were analyzed using a fixed-effects model according to the test of heterogeneity (chi-square = 6.05, *P* = 0.30, *I*^2^ *=* 17%). A significant decrease on BP was identified in favor of XZD therapy after treatment when compared with the antihypertensive drugs (RR = 1.32; 95% CI: 1.21 to 1.43, *P* < 0.00001) (Fig. 2C).

### Symptoms

**FIGURE 3:**
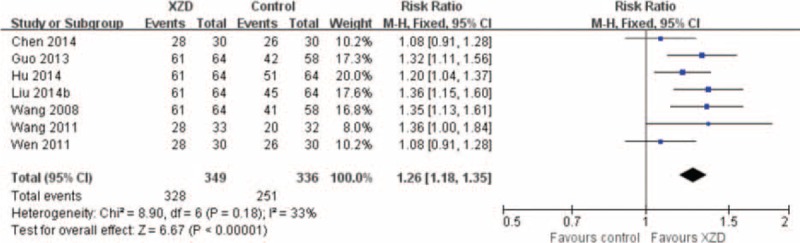
Forest plot of the comparison of XZD versus antihypertensive drugs for the outcome of symptoms. XZD = xuefu zhuyu decoction.

Seven studies assessed the effectiveness of XZD on the symptoms outcomes in comparison with antihypertensive drugs.^55–57,59,61,65,67^ There were 349 patients in the XZD groups and 336 patients in the antihypertensive drugs groups. A fixed-effects model was applied based on the test of heterogeneity (chi-square = 8.90, *P* = 0.18, *I*^2^ *=* 33%). The meta-analysis identified a significant improvement on the symptoms outcomes by XZD therapy compared with antihypertensive drugs (RR = 1.26; 95% CI: 1.18–1.35, *P* < 0.00001) (Fig. 3).

### Blood Lipids

**FIGURE 4:**
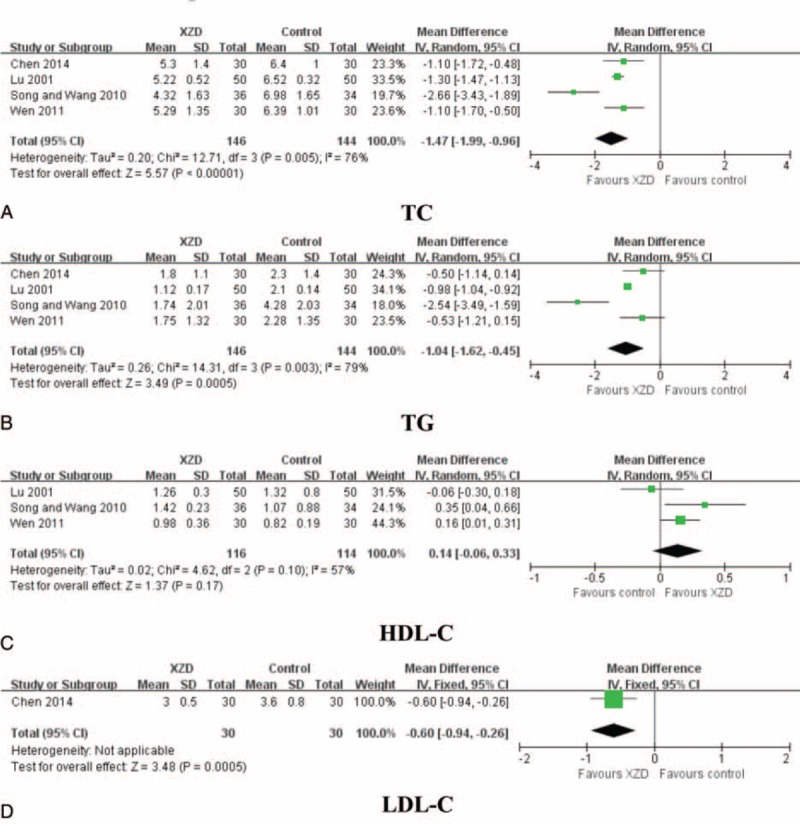
Forest plot of the comparison of XZD versus antihypertensive drugs for the outcome of blood lipids. A, TC; B, TG; C, HDL-C; and D, LDL-C. HDL-C = high-density lipoprotein cholesterol, LDL-C = low-density lipoprotein cholesterol, TC = total cholesterol, TG = triglycerides, XZD = xuefu zhuyu decoction.

Four studies used the outcomes of lipid profile parameters to evaluate the effectiveness of XZD in hypertensive patients, including total cholesterol (TC), triglycerides (TG), high-density lipoprotein cholesterol (HDL-C), and low-density lipoprotein cholesterol (LDL-C).^53,56,57,66^ There were 146 patients in the XZD groups and 144 patients in the antihypertensive drugs groups. Pooled analysis demonstrated a significant lipid-lowering effects of XZD therapy on TC (n = 4; WMD = −1.47 mmol/L; 95% CI: −1.99 to −0.96, *P* < 0.00001; heterogeneity: chi-square = 12.71, *P* = 0.005, *I*^2^ *=* 76%), TG (n = 4; WMD = −1.04 mmol/L; 95% CI: −1.62 to −0.45, *P* = 0.0005; heterogeneity: chi-square = 14.31, *P* = 0.003, *I*^2^ *=* 79%), and LDL-C (n = 1; WMD = -0.60 mmol/L; 95% CI: −0.94 to −0.26, *P* = 0.0005; heterogeneity: not applicable), beyond HDL-C (n = 3; WMD = 0.14 mmol/L; 95% CI: −0.06 to 0.33, *P* = 0.17; heterogeneity: chi-square = 4.62, *P* = 0.10, *I*^2^ *=* 57%) when compared with the antihypertensive drugs (Fig. 4).

### HCY

**FIGURE 5:**
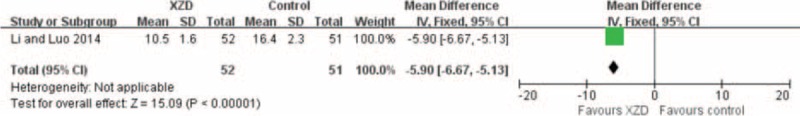
Forest plot of the comparison of XZD versus antihypertensive drugs for the outcome of HCY. HCY  = homocysteine, XZD = xuefu zhuyu decoction.

There was only 1 trial evaluating the effect of XZD with antihypertensive drugs alone on the outcome of serum HCY level.^60^ There were 52 patients in the XZD group and 51 patients in the antihypertensive drugs group. Pooled result was statistically significant in favor of XZD therapy (WMD = −5.90 μmol/L; 95% CI: −6.67 to −5.13, *P* < 0.00001; heterogeneity: not applicable) (Fig. 5).

### Hemorheology

**FIGURE 6:**
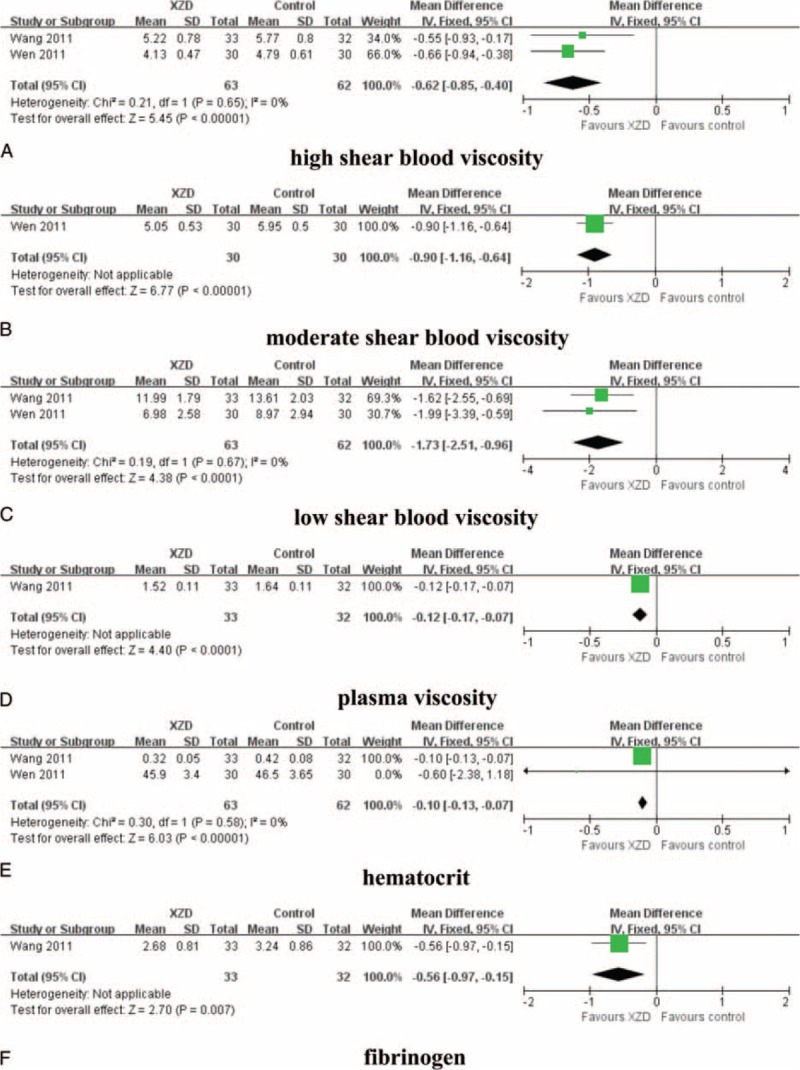
Forest plot of the comparison of XZD versus antihypertensive drugs for the outcome of hemorheology. A, high shear blood viscosity; B, moderate shear blood viscosity; C, low shear blood viscosity; D, plasma viscosity; E, hematocrit; and F, fibrinogen. XZD = xuefu zhuyu decoction.

The effects of XZD on the hemorheology outcomes, including high shear blood viscosity, moderate shear blood viscosity, low shear blood viscosity, plasma viscosity, hematocrit, and fibrinogen, were reported in 2 trials.^57,61^ There were 63 patients in the XZD groups and 62 patients in the antihypertensive drugs groups. The meta-analysis revealed significant effects of XZD for improving high shear blood viscosity (n = 2; WMD = −0.62 mPa/s; 95% CI: −0.85 to −0.40, *P* < 0.00001; heterogeneity: chi-square = 0.21, *P* = 0.65, *I*^2^ *=* 0%), moderate shear blood viscosity (n = 1; WMD = −0.90 mPa/s; 95% CI: −1.16 to −0.64, *P* < 0.00001; heterogeneity: not applicable), low shear blood viscosity (n = 2; WMD = −1.73 mPa/s; 95% CI: −2.51 to −0.96, *P* < 0.0001; heterogeneity: chi-square = 0.19, *P* = 0.67, *I*^2^ *=* 0%), plasma viscosity (n = 1; WMD = −0.12 mPa/s; 95% CI: −0.17 to −0.07, *P* < 0.0001; heterogeneity: not applicable), hematocrit (n = 2; WMD = −0.10 %; 95% CI: −0.13 to −0.07, *P* < 0.00001; heterogeneity: chi-square = 0.30, *P* = 0.58, *I*^2^ *=* 0%), and fibrinogen (n = 1; WMD = −0.56 g/L; 95% CI: −0.97 to −0.15, *P* = 0.007; heterogeneity: not applicable) (Fig. 6).

### IMT

**FIGURE 7:**
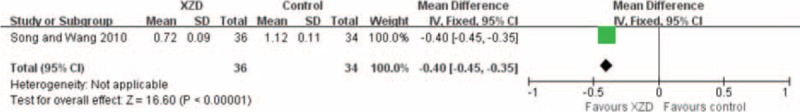
Forest plot of the comparison of XZD versus antihypertensive drugs for the outcome of IMT. IMT = carotid intima-media thickness, XZD = xuefu zhuyu decoction.

Only 1 trial tested the effect of XZD on the IMT outcome.^53^ There were 36 patients in the XZD group and 34 patients in the antihypertensive drugs group. Song and Wang^53^ reported a significant effect on the improvement of IMT when compared with antihypertensive drugs (WMD = −0.40 mm; 95% CI: −0.45 to −0.35, *P* < 0.00001; heterogeneity: not applicable) (Fig. 7).

### LVMI

**FIGURE 8:**
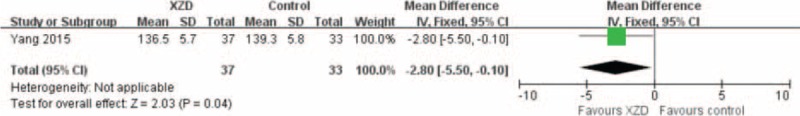
Forest plot of the comparison of XZD versus antihypertensive drugs for the outcome of LVMI. LVMI = left ventricular mass index, XZD = xuefu zhuyu decoction.

Only 1 trial evaluated the effectiveness of XZD on LVMI when compared with antihypertensive drugs alone.^58^ There were 37 patients in the XZD group and 33 patients in the antihypertensive drugs group. A significant improvement on LVMI in favor of XZD therapy was observed after treatment (WMD = −2.80 g/m^2^; 95% CI: −5.50 to −0.10, *P* = 0.04; heterogeneity: not applicable) (Fig. 8).

### Adverse Events

The outcome of adverse events was reported in 3 trials (3/15, 20.00%),^57,59,61^ whereas nothing was mentioned in the other 12 trials (12/15, 80.00%). Two studies reported that no adverse event was occurred in patients treated by either XZD or antihypertensive drugs.^57,59^ The third study reported that 3 patients with nausea (3/33, 9.09%) and 2 patients with dry cough (2/33, 6.06%) were identified in the XZD group, whereas 4 patients with dry cough (4/32, 12.50%) were observed in the fosinopril group. All of the reported adverse events were not severe and relieved without any treatment.

### Publication Bias

**FIGURE 9:**
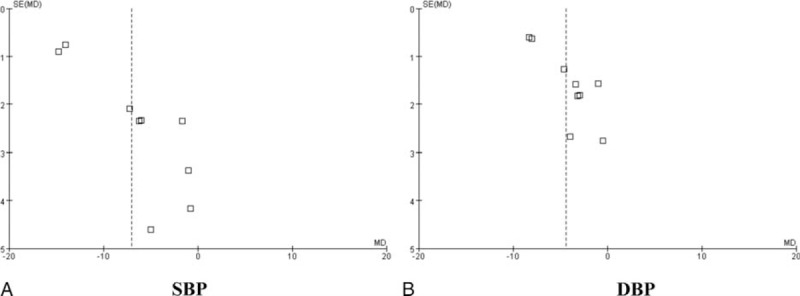
Funnel plot of the comparison of XZD versus antihypertensive drugs for the outcome of continuous BP. A, SBP and B, DBP. BP = blood pressure, DBP = diastolic blood pressure, SBP = systolic blood pressure, XZD = xuefu zhuyu decoction.

The funnel plot analyses of the 9 studies comparing XZD with antihypertensive drugs on SBP and DBP were generated to detect the potential publication bias. Significant asymmetry was manifested in the Figure 9A and B.

## DISCUSSION

### Summary of Evidences

Currently, there were clinical evidence ranged from case studies, case series, controlled trials to RCTs showing that XZD is effective in treating hypertensive patients; however, no high level of evidence such as systematic review or meta-analysis was provided for further recommendation. The purpose of this systematic review was to summarize the potential cardiovascular protective actions of XZD in patients with hypertension.

A total of 15 claimed RCTs involving 1364 hypertensive patients met the inclusion criteria in this review. In general, the pooled analyses of the current RCTs demonstrated a superior therapeutic effect of XZD as adjuvant therapy in treating hypertension. That is, XPAD is more effective in lowering BP, relieving symptoms, improving blood lipids, HCY, and hemorheology, and decreasing IMT and LVMI when compared with antihypertensive drugs alone.

The main therapeutic goal of treating hypertension are to not only reduce BP to the normal level, but also reverse cardiovascular risk factors, protect the target organs, and reduce mortality and cardiovascular events.^68–70^ This is a systematic review and meta-analysis on the potential role of XZD for hypertension. There were several strengths in this review. First, antihypertensive therapy is the cornerstone of hypertension treatment.^71^ On the basis of the guidelines on hypertension by the Eighth Joint National Committee, goal BP was <150/90 mm Hg in hypertensive persons aged ≥60 years, and goal DBP <90 mm Hg in hypertensive persons 30 to 59 years.^3^ Evidence also indicates that hypertensive patients could benefit from antihypertensive therapy when reaching the recommended threshold BP values. In our review, 9 trials (9/15, 60%) reported the outcomes on BP values and meta-analysis by subgroup showed that in hypertensive patients treated by XZD, the mean additional reduction in SBP was 6.99 mm Hg and DBP was 4.44 mm Hg. In the other 6 trials (6/15, 40%), the results also showed statistical significance compared with antihypertensive drugs alone. Our systematic review and meta-analysis was consistent with some prior reviews supporting use of traditional Chinese herbal formulae therapy for hypertension.^72–74^

Second, in some cases, the hypertension-related symptoms seriously troubled patients, although the elevated BP has been effectively controlled.^75^ According to the evaluation criterion in GCRNDTCM, these symptoms included headache, dizziness, insomnia, irritability, etc. We investigated the efficacy of XZD on the common symptoms in patients with hypertension in this study. Seven trials (7/15, 46.67%) were identified and the subgroup meta-analysis supported that XZD significantly improved symptoms in patients with hypertension; however, we should pay attention to that, an accurate TCM syndrome diagnosis is formed based on the collected symptoms and signs of the patients.^76,77^ Only 10 studies (10/15, 66.67%) reported the use of diagnostic criteria of TCM syndrome. As we know, a better therapeutic benefit might be achieved when the prescribed Chinese herbal medicine fit the TCM syndrome diagnosis.^78^ Therefore, we suggested that the theory of formula corresponding to syndrome in TCM should be reunderstood either in theory or in practice;^79^ and that both using and reporting the TCM syndrome diagnosis should be considered in further researches.

Third, the treatment goal of hypertension also includes managing the coexistent risk factors for cardiovascular disease together.^68,80,81^ The efficacy of XZD on blood lipids was evaluated in this study. A significant improvement on blood lipids was identified, with TC, TG LDL-C, and decreased by 1.47, 1.04, and 0.60 mmol/L, respectively. A clinically, but not statistically, significant increase in HDL-C was also observed by XZD therapy. HCY is regarded as a risk factor for hypertension and plays an important role in the development and progression of carotid atherosclerosis in hypertensive patients.^82,83^ Epidemiologic survey confirmed that high HCY level might increase the risk of hypertension.^84^ In this review, XZD significantly lowered the serum HCY level in hypertensive patients. Additionally, the hemorheology is an important biochemical index for diagnosing blood stasis syndrome and evaluating the therapeutic effects of PBCRBS-based herb and formulae in TCM.^31,85–87^ In our review, the hemorheology was significantly improved by XZD treatment comparing with the antihypertensive drugs alone. The results were consistent with previous meta-analysis of PBCRBS-based formulae on the outcomes of hemorheology.^88^ As only few studies provided data for blood lipids, HCY, and hemorheology, more clinical evidence are warranted to confirm the conclusions.

Fourth, an interesting finding of this review is the evaluation of XZD on target organ damage (TOD) in hypertensive patients. Long-term high BP induces vasculature, myocardium, and renal remodeling.^89^ Left ventricular hypertrophy, impaired renal function, and albuminuria are manifestations of TOD in hypertension, all of which are considered strong predictors for cardiovascular events and mortality.^90–94^ Therefore, current guidelines for the management of hypertension recommend that the preliminary evaluation of cardiovascular risks in hypertensive patient should focus on not only BP levels, but also TOD by measuring renal function, albuminuria, left ventricular hypertrophy, IMT, and pulse wave velocity .^1,69^ The effects of XZD on TOD were assessed in this systematic review and meta-analysis. A significant improvement on IMT and LVMI was identified in the XZD group compared with antihypertensive drugs alone.

Additionally, XZD treatment was well tolerated in the enrolled patients. No severe adverse events occurred in the XZD groups compared with the antihypertensive drugs groups. This systematic review suggested that XZD might be a safe TCM approach in managing hypertension; however, as only 3 trials reported the adverse events, it is still difficult to draw any definite conclusion.

## LIMITATIONS

Before accepting the above positive findings, the following limitations should also be considered. First, although comprehensive literature search was conducted in the 7 electronic databases, databases published in other languages except Chinese and English were not included in our study. Thus, a certain degree of potential selective bias might exist and some relevant publications of XZD might be missed.

Second, Vickers et al^95^ have pointed out that only positive results were produced in some countries. In our review, all of the 15 included studies were conducted in China and published in Chinese. Moreover, positive results were reported in most of the included studies and some negative results could not be reported. We understood that negative results were often difficult to be accepted in most Chinese journals currently. Thus, the efficacy of XZD for hypertension might be overestimated. Similar questions were also confronted in the previous published systematic reviews of Chinese herbal medicine.^96^

Third, we rigorously assessed the methodologic quality of the included trials based on the Cochrane Collaboration's tool. The methodologic quality is poor, which is the inherent shortcomings in primary studies. For example, all the included studies declared that, participants were randomized into the XZD group and antihypertensive drugs group; however, only 5 trials provided the adequate sequence generation and no trials reported the concealment of allocation. Inadequate reporting and poor methodologic design might weaken the strength and credibility of the clinical evidence of XZD in this review.

## CONCLUSION

In summary, XZD could improve BP, symptoms, blood lipids, HCY, hemorheology, IMT, and LVMI in hypertensive patients. Although some limitations such as potential selective bias and methodologic flaws might undermine the validity of positive findings, XZD is beneficial for hypertension treatment. From a clinical point of view, further RCTs with high-quality and long-term follow-up are recommended to generate high level of clinical evidence. Altogether, this systematic review and meta-analysis here provides an evidence-based approach to the management of hypertension and suggests XZD as a new candidate cardioprotective drug, which should be given priority for future preclinical and clinical studies.
